# Direct medical costs of cardiovascular diseases: Do cost components vary according to sex and age?

**DOI:** 10.1371/journal.pone.0311599

**Published:** 2024-10-10

**Authors:** Mahée Gilbert-Ouimet, Hélène Sultan-Taïeb, Ali Ben Charif, Chantal Brisson, Mathilde Lavigne-Robichaud, Alain Milot, Xavier Trudel, Éric Demers, Jason Robert Guertin

**Affiliations:** 1 Department of Health Sciences, Université du Québec à Rimouski, Lévis, QC, Canada; 2 Population Health and Optimal Health Practices Unit, CHU de Quebec Research Centre, Université Laval, Quebec City, QC, Canada; 3 Canada Research Chair in Sex and Gender in Occupational Health, Université du Québec à Rimouski, Lévis, QC, Canada; 4 School of Management, Université du Québec à Montréal (ESG-UQAM), Montréal, QC, Canada; 5 CubecXpert, Quebec City, Quebec, Canada; 6 Department of Social and Preventive Medicine, Université Laval, Quebec City, QC, Canada; 7 Department of Medicine, Université Laval, Quebec City, Canada; 8 Université Laval’s Research Center: The Tissue Engineering Laboratory (LOEX), Quebec City, Quebec, Canada; Faculty of Medicine, Saint-Joseph University, LEBANON

## Abstract

**Background:**

The aim was to estimate direct medical costs of men and women patients by age group related to cardiovascular diseases (coronary heart disease, strokes) in the province of Quebec, Canada from the economic perspective of the healthcare public system, encompassing five cost components: physician fees, hospitalization (hospital stay, intensive care stay), emergency visits and medication costs.

**Methods:**

This matched case-control study involved secondary data from a longitudinal cohort study (1997–2018) of 4584 white-collar workers. Participants were followed for a four-year period. We used an incremental cost method of difference-in-difference. Descriptive analyses using frequency counts, arithmetic means, standardized differences, chi-squared tests, and Student’s T-tests were performed. Direct medical costs were estimated using mean and 95% bootstrap confidence interval.

**Results:**

Direct medical costs per case were CAD $4970 [4344, 5595] for all in the first year after the event. For men patients, direct medical costs were $5351 [4649, 6053] and $4234 [2880, 5588] for women in the first year after the event, $221 [–229, 671] for men and $226 [–727, 1179] for women in the second year, and $11 [–356, 377] for men and $-24 [–612, 564] for women in the third year. This decrease was observed for both men and women, with higher costs for men. Within the first year, physician fees dominated CVD-associated costs among both men and women cases younger than 65. However, hospital stay represented the costliest component among cases aged 65 and older, incurring higher costs in women compared to men. In the subsequent years, the distribution of costs showed variations according to sex and age, with either medication costs or physician fees being the predominant components, depending on the specific subgroups.

**Conclusions:**

CVD-associated direct medical costs varied by components, sex, age, and follow-up years. Patients with CVD incurred more than twice the medical costs as compared to patients without CVD of same age and sex.

## Introduction

Cardiovascular diseases (CVD) constitute the leading cause of the disease burden and mortality among women and men in the world [1]. In Canada, heart diseases, which is the most frequent CVD, rank as the second-leading cause of death in men and women over the past 20 years [2], representing 24% of all deaths in 2020 [3]. This burden is expected to increase over the next decade, due to the aging of the population, improved survival of patients after a cardiovascular event and an increase in the prevalence of cardiovascular risk factors [1].

In Canada, circulatory system diseases (which include CVD) were the third costliest diseases in 2010 at an estimated 13 billion Canadian dollars (CAD) in direct medical costs [4]. Detailed cost-of-illness studies (COI) of CVD are needed to orient stakeholders toward efficient resource allocation for CVD prevention and treatment [5, 6]. These analyses estimate the relative contribution of the different components of an economic burden, allowing to tailor public policy measures according to current priorities for costs containment [7].

However, little is known about the direct medical costs of CVD in Canada and especially in Quebec. Provincial and territorial governments administer and deliver most of Canada’s health care services which explains variations in consumption of resources across provincial and territorial health care systems [8]. Moreover, the Canadian Management Information System Database contains financial and statistical operation information on public hospitals and regional health authorities across Canada [9], except for Quebec and Nunavut [9] where data is collected using a different standard [9]. Therefore, Quebec is one of the few Canadian provinces often excluded from estimates of the cost-of-illness and injury in Canada [4].

Previous Canadian studies examining the direct medical costs of CVD [10–15] fail to provide a global portrait. Indeed, most studies restricted their scope to a single CVD such as myocardial infraction [10, 11, 15], peripheral artery disease [11], and stroke [13, 14]. Furthermore, only four of these studies, examined these costs according to sex. All reported higher costs in men than women. Of these studies, the only two that considered patients’ age in addition to sex observed important subgroup differences, suggesting a steepest increase in costs due to aging in women than men [15], but lower costs in older women (>85 years-old) than in older men [14]. These findings highlight the importance for further sub-groups evaluations. Lastly, while direct medical costs tend to be higher in the first year of a CVD, costs occurring in the subsequent years also add pressure on the healthcare system and need to be documented, as recommended [7]. However, longitudinal estimations of these costs by sex and age are rare in Canada [11, 15].

This study sought to fill these gaps by estimating the direct medical costs of CVD (ischemic heart disease and stroke) in men and women patients, across age groups, from the economic perspective of the healthcare public system in the province of Quebec over a four-year period.

## Materials and methods

### Study design

This study followed an incidence-based matched case-control design from the economic perspective of the public healthcare system. This study followed the STrengthening the Reporting of OBservational studies in Epidemiology (STROBE) checklist [16], which is used to report observational research such as cohort, case-control, and cross-sectional studies. The Sex and Gender Equity in Research (SAGER) guidelines were also followed for reporting sex and gender information in study design, data analyses, results, and interpretation of findings [17].

Our study involved extensive participation of women (MGO, HST, MLR, and CB) and men (AM, XT, ABC, LM, and JRG) experts as members of the research team. Key stakeholder groups, including researchers and clinicians, were well represented among the team members.

### Cost components

Direct medical costs per individual encompassed five cost components (C):

- ***C***_**1**_**) Physician fees** referred to costs of inpatient and outpatient physician consultation visits (i.e., general practitioners and specialists).
- *C*_2_**) Hospitalization costs** included two components:
  ○ ***C***_**2*A***_**) Hospital stays** referred to costs related to the stays (e.g., laundry, food, administration, and maintenance), medical tests (all tests undertaken during stays), and the time spent by health professionals who were not physicians during inpatient stays.
  ○ ***C***_**2*B***_**) Intensive care costs.**
- ***C***_**3**_ **) Emergency visit costs.**
- ***C***_**4**_**) Medication costs**: referred to pharmaceutical dispensations. We subtracted the co-insurance amounts paid by patients to compute the actual amount paid by the public health insurance system for medications.

### Data sources

Data was extracted from complementary sources. The current study focused on participants of the PROspective Quebec (PROQ) Study on Work and Health [18], a longitudinal study initially including 9188 white-collar workers aged 18 to 65 years and employed in 19 public and semi-public organizations located in Quebec City, Canada. Participants were recruited in 1991–1993 and followed-up 8 years (1999–2001) and 24 years (2015–2018) later [18]. The general objective of the PROQ study was to extend knowledge of the adverse effects of psychosocial work stressors on the incidence of chronic diseases, including CVDs, among white-collar workers.

Data from two public healthcare administrative databases allowed to identify CVD cases among the PROQ Study participants: the RAMQ ([Régie de l’Assurance Maladie du Québec] healthcare insurance board) database and the MED‐ECHO database (Maintenance et exploitation des données pour l’étude de la clientèle hospitalière) compiling data related to stays in Quebec hospitals providing general and specialized care. Data on all-cause deaths was retrieved from the database of the Quebec Institute of Statistics.

Cost of medication were provided by the RAMQ database. RAMQ provides medication coverage through the public drug insurance program, known as the Public Prescription Drug Insurance Plan (PPIP), for eligible prescription drugs for Quebec residents who are not covered under a private drug insurance plan. Costs related to physician fees (inpatient, outpatient, intensive care acts, emergency visits) were also extracted from the RAMQ database and monetized according to the appropriate billing manual of the corresponding year [19]. Hospitalization costs were extracted from reports of the Finance Department of the *CHU de Québec—*Université Laval, which provided average daily costs for hospital stays and emergency visits for each year of the follow-up. Lastly, the number of days of hospital stay, intensive care and emergency visit days per case were extracted from the MED-ECHO database.

### Study participants

Eligible participants were CVD cases and matched controls from the PROQ Study [18]. More specifically, ***cases*** that occurred between 1 January 1998 and 31 December 2018 were eligible. Cases were identified with data on CVD events retrieved from their patient files provided by the RAMQ and MED-ECHO databases, using previously validated algorithms for coronary heart disease and for stroke [20, 21] (**S1 File**). Each case corresponded to a participant having a first CVD event, this event corresponding to the index date. Each case was followed for a four-year period in parallel to its matched control(s): one year prior to a first CVD event (Year 0), the first, second and third years after the event (Year 1, Year 2, Year 3). Right censoring occurred when a case died or after a four-year follow-up, whichever came first. Cases who died on the first day of their cardiovascular event or during their first hospitalization (from any cause) were excluded (n = 42). Cases with CVD recurrence or concomitant diseases were not censored, as all direct medical costs incurred following the first CVD event were included in calculations.

***Controls*** were participants of the PROQ study free of CVD events before and throughout the follow-up years. Every case was matched with all eligible controls sharing the same sex and age (±2 years caliper), which are two potentially confounding factors [22]. The matching procedure did not include other cardiovascular risk factors, as cases and controls with similar risk profiles could have led to an underestimation of the costs associated with CVD. To be eligible, controls had to be followed at the index date of their corresponding case and for at least as long as the case. Therefore, cases and controls had the same index dates.

### Covariates

Socio-demographic characteristics (age at the index date for the cases and controls, sex at birth, education level, household income, and marital status) and other cardiovascular risk factors (smoking status, body mass index, leisure-time physical activity, and alcohol intake) collected at the baseline (1991–1993) of PROQ study were used as covariates [18].

### Statistical analysis

Overall, descriptive analyses were performed and direct medical costs were estimated. For the costs of hospitalization stay, intensive care, or emergency visits, the annual number of days of stay of each participant was multiplied by the average daily stay costs of the same year (obtained from the CHU de Québec). For descriptive analyses, we computed frequencies (n, %) for categorical characteristics and arithmetic means (averages) and standard deviation (SD) for continuous characteristics. Chi-squared tests were applied for categorical characteristics and Student’s T-tests for continuous characteristics to compare case and control patients at baseline. All tests were two-sided, and the statistical significance threshold was considered to be p-value <0.05. For all characteristics, standardized differences were computed, which refer to the difference between means of the continuous measure or proportions of categorial measures in the case group and in the control group divided by the standard error of the measure [23, 24]. For estimation of direct medical costs, arithmetic means and 95% bootstrap confidence interval (CI) using 1000 iterations [25] were computed. Direct medical costs were calculated and reported per cost component separately and globally in the first, second, and third year. To account for the direct medical costs of right-censored subjects, the inverse probability weighting method was used [26]. Thus, the cumulative direct cost of each case who died or reached the full period of observation represented not only the direct cost of that participant, but also the censored cases that would have been observed had there been no censoring. Costs for women and men across three age groups (<55 years, 55–64 years and ≥65 years) were compared over the follow-up period. All costs were in Canadian dollars (CAD) and adjusted for the year 2019 based on the all-item Canadian consumer price index [27]. All analyses were performed with SAS 9.4 software.

Incremental costs for each case were defined as costs in the given year minus costs in the year before the event. Before-after incremental costs for a case *i* (for *i* = 1,…,*n*; *n*≥1) in the year *Y*_*t*_ (for *t* = 1,2,3) was defined as:

$${Before-after\;incremental\;cost}_{Y_{t}}^{case\;i}=\sum_{k=1}^{5}{(C_{k}}_{Y_{t}}^{Case\;i}-{C_{k}}_{Y_{o}}^{Case\;i})$$ (1)

where:

- *Y*_*t*_ is the year of interest (i.e., first, second, or third year after the event);
- *Y*_0_ is the year leading up to the event;
- *C*_*k*_ (for *k* = 1,2,3,4,5) is the cost component: *C*_1_) physician fees, *C*_2_) hospitalization costs, *C*_3_) intensive care costs, *C*_4_) emergency visit costs, and *C*_5_) medication costs);
- ${C_{k}}_{Y_{t}}^{Case\;i}$ is the cost component *C*_*k*_ of the case *i* for the year *Y*_*t*_ and ${C_{k}}_{Y_{o}}^{Case\;i}$ the cost component *C*_*k*_ of the case *i* for the year *Y*_0_.

CVD-associated direct medical costs were calculated using a difference-in-difference costing method [28], in which the difference between direct medical costs for cases and controls was regarded as being attributable to the CVD [29–31]. Thus, CVD-associated medical costs of a case were the costs of the case minus the costs of its matched controls. Mathematically, CVD-associated direct medical costs for a Case *i* matched to Controls *i* (for *i* = 1,…,*n*; *n*≥1) in year *Y*_*t*_ (for *t* = 1,2,3) was defined as:

$${CVD-associated\;cost}_{Y_{t}}^{Case\;i}=\sum_{k=1}^{5}\left[{(C_{k}}_{Y_{t}}^{Case\;i}-{C_{k}}_{Y_{o}}^{Case\;i})-({AVE(C_{k}}_{Y_{t}}^{Controls\;i})-{{AVE(C}_{k}}_{Y_{0}}^{Controls\;i}))\right]$$ (2)

With ${{AVE(C}_{k}}_{Y_{t}}^{Controls\;i})$, the average cost component *C*_*k*_ of all matched controls of the case *i* for the year *Y*_*t*_, and ${{AVE(C}_{k}}_{Y_{0}}^{Controls\;i})$ for the year *Y*_0_.

### Ethics approval and consent to participate

This project was approved by the ethics committee of the Research Center of the CHU of Quebec ([Centre Hospitalier Universitaire], University hospital center)–Université Laval (#2012–1674, DR-002-1409).

## Results

### Characteristics of participants

We selected a total of 4584 eligible participants: 1543 cases matched to 2810 controls (**S2 File**). Each case was matched to at most two controls: 82.1% with two controls and 28.9% with one control. There was no significant difference between cases and controls in terms of sex at birth (*p* = 0.301), household income (*p* = 0.053), and alcohol intake (*p* = 0.121) (**S3 File**). Compared with controls, cases were older, less educated, smokers, overweight, and less active (*p*<0.05).

### Before-after incremental costs

**Table 1** outlines the average before-after incremental costs per case of CVD for the follow-up period after the first CVD event: CAD $5180 [4567, 5793] for the first year, $582 [171, 994] for the second year, and $465 [212, 719] for the third year. Physician fees ($1818 [1635, 2000]) constituted the highest direct cost component in the first year. Medication costs was the highest cost component in the second ($247 [143, 351]) and third year ($318 [222, 414]) after the event. Emergency visit costs constituted only small proportions of these costs in the first ($79 [56, 102]), second ($-46 [–64, –27]), and third ($-34 [–53, –15]) year after the event.

**Table 1:** Incremental costs before-after a first cardiovascular event and CVD-associated costs per case (in 2019 Canadian $).

Cost component		Year 1 (*n* = 1472) (*mean* [95%*CI*])		Year 2 (*n* = 1472) (*mean* [95%*CI*])		Year 3 (*n* = 1472) (*mean* [95%*CI*])	
		Incremental costs	CVD-associated costs	Incremental costs	CVD-associated costs	Incremental costs	CVD-associated costs
*C*_1_) Physician		1818 [1635, 2000]	1753 [1567, 1939]	50 [–57, 157]	-43[–161, 75]	17[–64, 98]	-116 [–212, –20]
*C*_2_ *) Hospitalization*		*2994[2538*, *3450]*	*2879 [2415*, *3344]*	*331 [27*, *635]*	*138 [–191*, *466]*	*164 [–3*, *331]*	*3[–201*, *208]*
	*C*_2*A*_) Hospital stay	1792 [1492, 2091]	1685[1377,1993]	183 [7, 360]	-1 [–229, 228]	128 [–19, 275]	-41 [–228,147]
	*C*_2*B*_) Intensive care	1203 [962, 1444]	1194[953, 1436]	148 [–84, 380]	139 [–93, 371]	36 [–18, 90]	44 [–11, 99]
*C*_3_) Emergency visit		79 [56, 102]	63 [38, 88]	-46 [–64, –27]	-55 [–76, –35]	-34 [–53, –15]	-59 [–81, –38]
*C*_4_) Medication		289 [211, 367]	274 [194, 354]	247 [143, 351]	183 [74, 292]	318 [222, 414]	171 [43, 298]
**Total**		5180 [4567, 5793]	4970 [4344, 5595]	582 [171, 994]	223 [–223, 669]	465 [212, 719]	-1 [–316, 313]

CI: confidence interval.

### CVD-associated direct medical costs

About 96% of CVD-associated costs per case were related to the first year after the event (mean [95% CI]: $4970 [4344, 5595]), followed by 4% the second year ($223 [–223, 669]) (**Table 1**). These costs were dominated by physician fees in the first year ($1753 [1567, 1939]) and medication costs in the second ($183 [74, 292]) and third year ($171 [43, 298]) after the event.

### CVD-associated costs among men and women

**Table 2** outlines that CVD-associated direct medical costs per case for men patients were $5351 [4649, 6053] and $4234 [2880, 5588] for women in the first year post-event, $221 [–229, 671] for men and $226 [–727, 1179] for women in the second year, and $11 [–356, 377] for men and $-24 [–612, 564] for women in the third year. For men patients, these costs were dominated by physician fees in the first year ($1980 [1751, 2209]), whereas for women hospital stay constituted the highest cost component ($1563 [998, 2128]). For men, medication costs were the highest component in the second year ($233 [156, 310]), whereas for women intensive care costs were the highest component ($320 [–351, 992]). For men and women, the highest cost component was medication in the third year.

**Table 2:** CVD-associated direct medical costs per case by sex (in 2019 Canadian $).

Cost component		Year 1 (*mean* [95%*CI*])		Year 2 (*mean* [95%*CI*])		Year 3 (*mean* [95%*CI*])	
		Men (*n* = 969)	Women (*n* = 503)	Men (*n* = 969)	Women (*n* = 503)	Men (*n* = 969)	Women (*n* = 503)
*C*_1_) Physician		1980 [1751, 2209]	1315 [941, 1689]	-44 [–176, 88]	-41 [–260, 178]	-104 [–216, 7]	-138 [–313, 37]
*C*_2_ *) Hospitalization*		*2977 [2483*, *3471]*	*2691 [1669*, *3714]*	*79 [–262*, *419]*	*252 [–444*, *950]*	*-4 [–232*, *225]*	*17 [–393*, *427]*
	*C*_2*A*_) Hospital stay	1748[1373, 2124]	1563 [998, 2128]	34 [–284, 352]	-68[–329, 194]	-35 [–249, 180]	-52 [–414, 309]
	*C*_2*B*_) Intensive care	1229 [1017, 1440]	1128 [534, 1723]	45[–31, 120]	320[–351, 992]	31 [–12, 74]	69 [–67, 205]
*C*_3_) Emergency visit		82 [50, 114]	29 [–16, 73]	-47 [–71, –21]	-74 [–110, –37]	-62 [–87, –37]	-54 [–93, –15]
*C*_4_) Medication		312 [203, 421]	199 [(80, 317]	233 [156, 310]	88 [–197, 373]	181 [23, 340]	151 [–62, 364]
**Total**		5351 [4649, 6053]	4234 [2880,5588]	221 [–229, 671]	226 [–727, 1179]	11 [–356, 377]	-24 [–612, 564]

CI: confidence interval.

### CVD-associated costs by sex and age

**Table 3** shows that the distribution of costs varied according to sex and age. Overall, CVD-associated direct medical costs occurring in the first year post-event were dominated by physician fees. However, there were exceptions among patients over the age of 65, where hospital stay was the highest cost component in women ($3005 [1406, 4605]). In the first year, physician fees were higher in men than women, with a particularly pronounced difference in patients aged 65 and older. Additionally, during this period, hospital stay and intensive care costs were higher for women than men in the 65 and older age group.

**Table 3:** CVD-associated costs per case by sex and age after a first cardiovascular disease event (in 2019 Canadian $).

Cost component	Year 1 (*mean* [95%*CI*])		Year 2 (*mean* [95%*CI*])		Year 3 (*mean* [95%*CI*])	
	Men	Women	Men	Women	Men	Women
Aged under 55	*n* = 220	*n* = 147	*n* = 220	*n* = 147	*n* = 220	*n* = 147
*C*_1_) Physician	1548 [1175, 1921]	1282 [796, 1767]	127 [–73, 328]	-175 [–349, –1]	59 [–111, 229]	-104 [–406, 199]
*C*_2_ *) Hospitalization*	*2403 [1659*, *3148]*	*1777 [1069*, *2484]*	*133 [–321*, *587]*	*-70 [–293*, *153]*	*126 [–158*, *409]*	*-19 [–449*, *411]*
*C*_2*A*_) Hospital stay	1073 [719, 1427]	911 [491, 1331]	3 [–323, 329]	-60 [–274, 154]	58 [–164, 281]	4 [–423, 431]
*C*_2*B*_) Intensive care	1330 [796, 1865]	866 [451, 1280]	130 [–89, 348]	-10 [–44, 25]	68 [–41, 176]	-23 [–56, 9]
*C*_4_) Emergency visit	94 [54, 133]	49 [–11, 109]	-14 [–53, 24]	-58 [–106, –10]	-33 [–71, 5]	-11 [–83, 61]
*C*_5_) Medication	40 [11, 70]	-8 [–97, 81]	26 [–21,72]	60 [–46, 165]	28 [–29, 85]	34 [–51, 119]
**Total**	4086 [3002, 5169]	3100 [1922, 4278]	271 [–351, 893]	-243 [–614, 127]	180 [–272, 631]	-100 [–824, 624]
**Aged 55–64**	*n* = 395	*n* = 197	*n* = 395	*n* = 197	*n* = 395	*n* = 197
*C*_1_) Physician	1637 [1323, 1952]	928 [387, 1470]	-270 [–445, –96]	-160 [–471, 152]	-354 [–532, –175]	-152 [–423, 119]
*C*_2_ *) Hospitalization*	*2366 [1754*, *2977]*	*1654 [694*, *2615]*	*-188 [–621*, *244]*	*5 [–369*, *379]*	*-377 [–797*, *43]*	*443 [–269*, *1155]*
*C*_2*A*_) Hospital stay	1296 [855, 1738]	885 [309, 1462]	-159 [–563, 244]	7 [–292, 305]	-370 [–762, 22]	238 [–285, 761]
*C*_2*B*_) Intensive care	1069 [804, 1334]	769 [230, 1308]	-29 [–114, 57]	-2 [–169, 166]	-7 [–76, 63]	205 [–114, 524]
*C*_3_) Emergency visit	73 [26,120]	6 [–65, 76]	-70 [–106, –33]	-78 [–132, –24]	-85 [–125, –44]	-77 [–131, –24]
*C*_4_) Medication	172 [84, 261]	139 [–13, 290]	175 [54, 295]	197 [–110, 505]	126 [–89, 341]	240 [–234, 714]
**Total**	4248 [3346, 5150]	2727 [1337, 4117]	-354 [–943, 236]	-36 [–808, 737]	-689 [–1301, –78]	453 [–519, 1425]
**65 and older**	*n* = 354	*n* = 159	*n* = 354	*n* = 159	*n* = 354	*n* = 159
*C*_1_) Physician	2632 [2114, 3150]	1826 [960, 2691]	102 [–164, 368]	230 [–304, 765]	72 [–123, 267]	-153 [–482, 176]
*C*_2_ *) Hospitalization*	*4015 [2898*, *5133]*	*4822 [1894*, *7750]*	*343 [–373*, *1060]*	*859 [–1285*, *3003]*	*332 [–33*, *697]*	*-477 [–1332*, *377]*
*C*_2*A*_) Hospital stay	2672 [1791, 3554]	3005 [1406, 4605]	269 [–421, 958]	-166 [–901, 569]	282 [–73, 637]	-464 [–1298, 370]
*C*_2*B*_) Intensive care	1343 [937, 1749]	1817 [105, 3529]	75 [–67, 216]	1025 [–1082, 3133]	50 [–11, 111]	-13 [–198, 172]
*C*_3_) Emergency visit	84 [20, 147]	38 [–55, 130]	-39 [–89, 10]	-82 [–163,– 2]	-56 [–103, –9]	-64 [–144, 16]
*C*_4_) Medication	638 [359, 917]	464 [156, 772]	426 [269, 584]	-22 [–848, 805]	339 [–25, 702]	149 [–213, 511]
**Total**	7369 [5758, 8981]	7149 [3381, 10917]	832 [–88, 1752]	985 [–1838, 3809]	687 [30, 1343]	-545 [–1759, 668]

CI: confidence interval.

In the second year, intensive care costs dominated for women aged 65 and older, while medication was the highest cost component for women under that age. In men, intensive care and physician fees costs were the costliest components under 55 years-old, whereas medication was the predominant component beyond that age. In the third year, medication costs was the highest cost component for all groups except for men under 55 years, where intensive care costs were the highest, closely followed by physician fees and hospital stays. It is noteworthy that, during this period, for women aged between 55 and 64 years, CVD-associated costs related to hospital stays were nearly identical to medication costs.

## Discussion

### Main results

In this matched case-control study, we described direct medical costs associated with a first CVD event from 1997 to 2018, from the economic perspective of the healthcare public system. CVD-associated direct medical costs were highest in the first year after the event and decreased over time. This trend was observed in both men and women patients. Examining costs across age groups in addition to sex brought forth differences between subgroups. In the first year following the event, physician fees dominated CVD-associated costs among both men and women cases younger than 65, with higher costs in men. However, among cases aged 65 and older, hospital stay represented the costliest component, incurring higher costs in women compared to men. Although generally smaller, variations according to age and sex were also observed in the distribution of costs in the subsequent years, with either intensive care, medication or physician fees being the predominant cost components, depending on the specific subgroups. Overall, patients with cardiovascular diseases incurred more than twice the medical costs as compared to a patient without cardiovascular diseases of same age and sex.

### Comparison with the literature

Comparing our results with the literature poses several challenges. First, the cases included in our study were defined by the incidence of a first CVD event. Not all participants had a hospital stay following their event, whereas patient hospitalization is often used as an inclusion criterion in studies on CVD direct medical costs [10, 13–15]. This may have contributed to lower estimates in our study, as a broader range of case severity were included. Second, since previous Canadian studies used a cost-specific method [10–15], which is not comparable to the difference-in-difference method applied in the current study. The cost-specific method provides an inventory of every item (e.g., a given medication, a hospital stay, a physician consultation) in order to attribute their costs to different groups of diseases and retain only those specific to the disease under study. While this method tends to be detailed and precise, it likely underestimates total direct medical costs of illnesses. Applying a difference-in-differences method using a matched case-control design has been suggested as a more valid approach [7] by quantifying the excess costs of the disease, including both disease-specific costs and comorbidity costs [32]. This method also contributes to control for selection biases through a comparison of changes in costs occurring over time between a group affected by a disease and a control group, thereby accounting for pre-existing differences.

Consistently with previous findings, almost all the direct medical costs associated with CVD in the current study occurred in the first year after the event, then decreased over time for both men and women. Clear decreases in direct medical costs after a first stroke [30, 33, 34] or ischemic heart disease [10, 12, 35] have previously been reported. CVDs are chronic diseases and tend to manifest themselves more aggressively at the beginning with a higher risk of adverse outcomes. This phase requires more spendings related to early consultations, prompt medications, patient regular monitoring, and longer length of hospitalization stay to reduce damage and other complications [29].

Also consistently with the literature [10, 12, 14, 15], higher costs were observed for men than women in the current study. However, for women aged 65 and older during the first year following a CVD event, costs were dominated by hospital stay costs and this cost component, as well as intensive care costs, were higher for women than for men of the same age group. Given the documented pitfalls of CVD detection and treatment in women [36], this might lead older women to need more secondary compared to primary care than men. Indeed, when considering patients age, the lack of recognition of sex differences can pose serious threats to women cardiovascular health. Indeed, while the incidence of CVD is known to be higher in middle-aged men than women, aging women have a similar level of risk once the estrogen protection diminishes from menopause [37]. Then, women are facing poorer CVD outcomes than men [38, 39]. Cardiovascular research examining sex differences is still needed, as women have been underrepresented within the body of research destined to orient the detection, prevention and management of CVD [36, 40]. In turn, they do not benefit from optimal prevention strategies and care [41, 42]. Further examinations of sex and age differences in the CVD-attributable costs, and their underlying mechanisms, are required to improve prevention and achieve better equity in health [43], especially during the first year following the event.

### Limitations

Caution should be exercised when interpreting the implications of our findings. Our results are conservative for two main reasons. First, the costs of outpatient visits to health professionals other than physicians (e.g., a psychologist, a social worker, massage therapist, nursing practitioner) in public healthcare institutions could not be considered due to lack of available data. Also, the costs associated with public nursing homes and long-term care establishments, other than hospitals, are not available in the MED-ECHO database. However, this likely had a limited impact on our estimates, as expenses for long-term care facilities constitute a smaller portion of total public health expenditures in Quebec (and Canada) compared to other countries [44]. Second, medication costs in our study corresponded to ambulatory outpatient medication as reimbursed by the RAMQ to individuals enrolled in the Quebec public drug insurance plan (the alternative for medication reimbursement being private insurance plans). Moreover, medication received during hospitalization stays were also considered but under the average daily costs of hospital stays. Therefore, inpatient medication costs were included in hospital stays costs and not medication costs in our study. Third, unit costs for hospital stay, intensive care and emergency visits were based on an average amount per day of stay for all patients and on a single healthcare center in Quebec City. Although most patients in our cohort were from this city, unit costs for these healthcare resources could differ between healthcare centers across the province [45]. Fourth, our results correspond to an average estimate of costs over a long period of time (1997–2018) and the amount as well as the distribution of cost components have changed over this period of time because of changes in clinical practice over the last 20 years [10]. Nevertheless, the primary contribution of the current study consisted in providing one of the rare longitudinal estimations of CVD direct medical costs by sex and age in Canada. Period-analyses across sub-groups of our cohort would have relied on a small number of cases, especially at the beginning of the follow-up, as the included participants were younger workers. Identifying these periods would also have been challenging as guidelines and practices changed on various dates for the different CVD examined. It is also noteworthy that findings across sub-groups considering simultaneously sex and age need to be replicated due to the limited number of cases. Finally, the PROQ study includes women and men working in a wide range of white-collar jobs covering a diversity of socioeconomic statuses (S3 File). While white-collar workers constitute the largest segment of the Canadian working population (52.7%) [46], the external validity of our estimates warrant caution due to a potential healthy worker effect [47]. Nevertheless, it is worth mentioning that the prevalence of CVD (8%) was comparable to that observed in Canadian and American population surveys of a comparable age structure [48, 49]. Also, the prevalence of major CVD risk factors, such as smoking (21%) and leisure-time physical activity (21%), was comparable to that observed in a representative sample of the Quebec population [50].

### Strengths

A systematic review of studies on CVD direct costs reported that only four of the 82 included studies met several methodological quality criteria [51], which were met by the present study.: 1) estimating costs per case longitudinally over a period of four years; 2) distinguishing different cost components on a yearly basis based on a bottom-up approach [6]; 3) comparing patients who experienced CVD events with matched cohorts of patients without CVD events; 4) using a difference-in-difference method allowing to identify yearly costs as a difference between the year before and years after a CVD for a given case along with associated costs as a difference between cases and controls. This method covers the costs of comorbidities [32], prevents the misallocation of costs associated to CVD and corresponds to best practices according to the literature [7]. Lastly, our study considered first year costs and costs in subsequent years along with sex and age to identify sub-group differences in direct medical costs associated with CVD.

## Conclusion

Based on a longitudinal analysis of a cohort database, our findings highlight that direct medical costs associated with a first CVD are high, especially during the first year, which emphasizes the need for primary prevention. Our findings by cost components and sub-groups of patients provide new insight to policymakers, clinicians, and quality improvement healthcare services to better understand resource allocation related to CVD according to sub-groups of patients. These results also emphasize differences between men and women patients in cost components during the first year following a CVD event and may have implications for action regarding the management of cardiovascular diseases by the healthcare system. Further investigations are required for a deeper analysis of potential inequities between men and women across age groups in the detection and treatment of CVD.

## Supporting information

S1 File Algorithms. (DOCX)

S2 File Sampling process. (TIF)

S3 File Characteristics of participants. (DOCX)
