# Supplementary material for: Direct medical costs of cardiovascular diseases: Do cost components vary according to sex and age?
Source: PLoS One. 2024 Oct 10;19(10):e0311599. doi: 10.1371/journal.pone.0311599 (PMC11466411; doi:10.1371/journal.pone.0311599)
Supplement: S1 File — (DOCX) [file pone.0311599.s001.docx]

**S1 File: Algorithms for identifying first cardiovascular events**

| Cardiovascular Disease | Definition | Algorithm | Code and procedure |
| --- | --- | --- | --- |
| Ischemic heart diseases (IHD) | Ischemia refers to inadequate blood circulation to a local area due to blockage of the blood vessels supplying the area. Ischemic means that an organ (e.g., the heart) is not getting enough blood and oxygen. Ischemic heart disease (also known as coronary heart disease or coronary artery disease) is the term given to heart problems caused by narrowed heart (coronary) arteries that supply blood to the heart muscle(70) | [Death from IHD] **OR**  [One or more hospitalizations with an ICD-9(-CM) or procedure code (CCP) for ischemic heart diseases, any time during the period of selection, or equivalent ICD-10(-CA) and procedure code (CCI)] **OR**  [Two or more physicians claimed with ICD-9(-CM) or ICD-10(-CA) codes for ischemic heart diseases, within one year. Consultations in the same day are counted only if performed by different doctors. The event date was determined by the date of the second physician's visit for ischemic heart diseases] | ICD-9 codes:   - 410 - 414   ICD-10 codes:   - I20-I25   CCP codes:   - 48.02, 48.03, 48.09, 481.   CCI codes:   - 1.IJ.50, 1.IJ.57.GQ, 1.IJ.76. |
| Stroke | A stroke (also known as cerebrovascular disease or brain attack) occurs when something blocks blood supply to part of the brain or when a blood vessel in the brain bursts. Strokes happen in two ways: 1) A blocked artery can cut off blood to an area of the brain. This is known as an ischemic stroke and 85% of strokes are of this type; 2) The second type of stroke happens when a blood vessel can leak or burst. So the blood spills into the brain tissue or surrounding the brain. And this is called a hemorrhagic stroke. | [Death from stroke] **OR**  [One or more hospitalizations with evidence of a stroke any time during the period of selection] **OR**  [Two or more physicians claimed with evidence of a stroke within one year, whichever comes first. Consultations in the same day are counted only if performed by different doctors. The event date was determined by the date of the second physician's visit for stroke (including transient ischemic attack)] | ICD-9 codes:   - 362.3, 430, 431, 432, 434.X*, 435.X*, 436.   ICD-10 codes:   - I60.X*, I61.X*, I63.X* (excluding I63.6), I64, H34.0, H34.1, G45.X* (excluding G45.4). |
| Abbreviations: ICD: International Classification of Diseases; ICD-9-CM: International Classification of Diseases (ICD), 9^th^ Revision, Clinical Modification (CM); ICD-10-CA: International Statistical Classiﬁcation of Diseases and Related Health Problems, 10th Revision, Canada; CCP: Canadian Classification of Diagnostic, Therapeutic, and Surgical Procedures (CCP); CCI: Canadian Classification of Health Interventions;  Notes: *Unless otherwise noted, ".X" indicates that all variations of the diagnostic code are included. | | | |
