# Supplementary figures and images for: Direct medical costs of cardiovascular diseases: Do cost components vary according to sex and age?
Source: PLoS One. 2024 Oct 10;19(10):e0311599. doi: 10.1371/journal.pone.0311599 (PMC11466411; doi:10.1371/journal.pone.0311599)

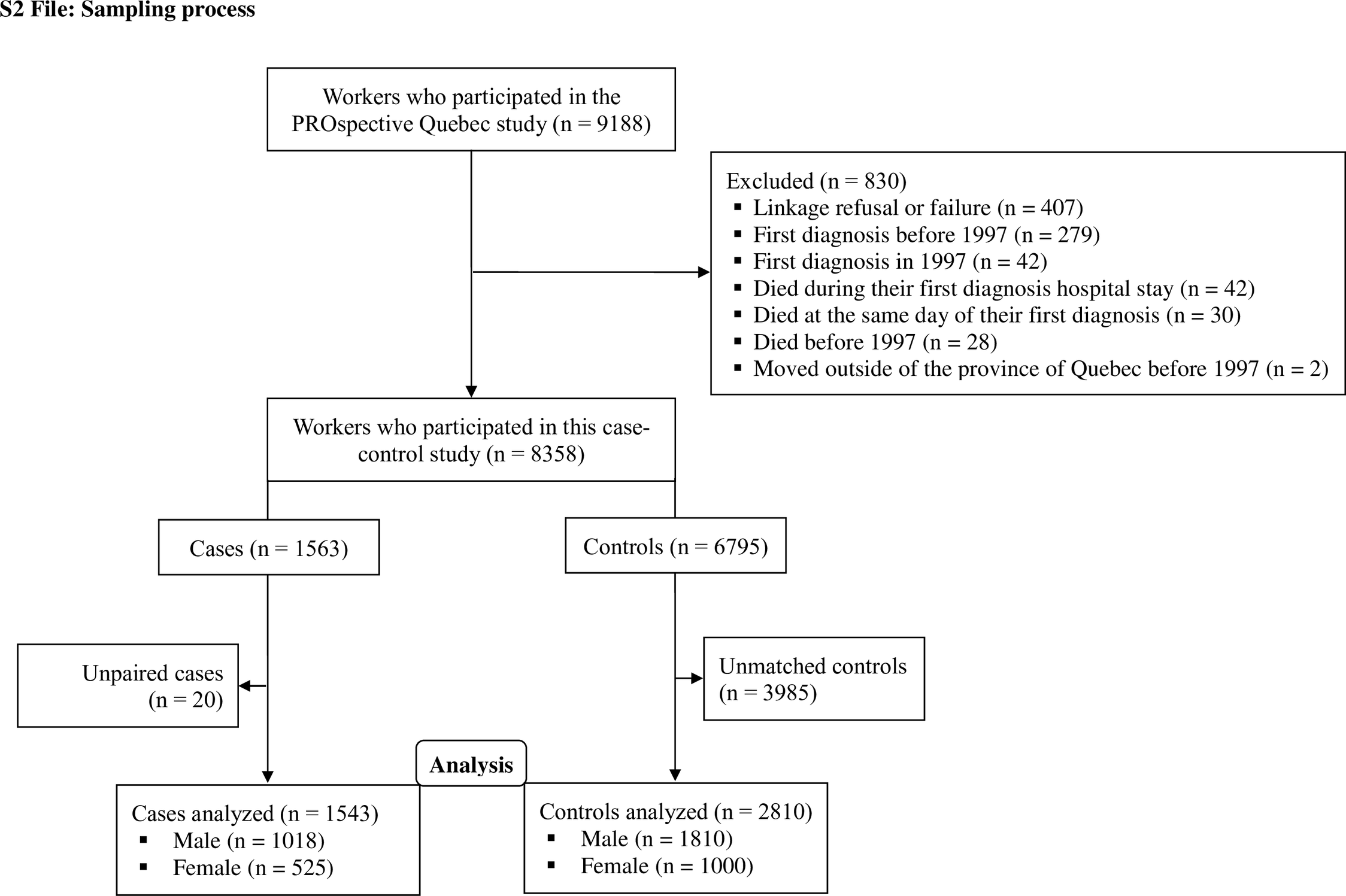

Supplement: S2 File — (TIF) [file pone.0311599.s002.tif]
