# Supplementary material for: Direct medical costs of cardiovascular diseases: Do cost components vary according to sex and age?
Source: PLoS One. 2024 Oct 10;19(10):e0311599. doi: 10.1371/journal.pone.0311599 (PMC11466411; doi:10.1371/journal.pone.0311599)
Supplement: S3 File — (DOCX) [file pone.0311599.s003.docx]

**S3 File: Characteristics of participants at the baseline of the PROQ study**

|  | | | **Cases (n = 1543)** | | **Controls (n = 2810)** | | ***P* value** | **Standardized difference** |
| --- | --- | --- | --- | --- | --- | --- | --- | --- |
| **Continuous characteristics** | | | *Mean* | *Standard deviation* | *Mean* | *Standard deviation* | *Student's t test* | % |
|  | Age in years at first event | | 61.2 | 9.1 | 60.1 | 8.7 | <0.001 | 11.6 |
| **Categorical characteristics** | | | *n* | *% column* | *n* | *% column* | *Chi-square test* |  |
|  | Age in years at first event^1^ | |  |  |  |  | 0.008 |  |
|  |  | Under 55 | 370 | 24.0 | 749 | 26.7 |  | -6.2 |
|  |  | 55 – 64 | 615 | 39.9 | 1172 | 41.7 |  | -3.8 |
|  |  | 65 or older | 558 | 36.2 | 889 | 31.6 |  | 9.6 |
|  | Sex at birth^1^ | |  |  |  |  | 0.301 |  |
|  |  | Male | 1018 | 66.0 | 1810 | 64.4 |  | 3.3 |
|  |  | Female | 525 | 34.0 | 1000 | 35.6 |  | -3.3 |
|  | Education level^1,2^ | |  |  |  |  | <0.001 |  |
|  |  | Less than collegial | 483 | 31.6 | 695 | 24.9 |  | 14.8 |
|  |  | Collegial | 390 | 25.5 | 717 | 25.7 |  | -0.5 |
|  |  | University | 656 | 42.9 | 1376 | 49.4 |  | -13.0 |
|  | Household income (CAD)^1,2^ | |  |  |  |  | 0.053 |  |
|  |  | Under 50 000 | 536 | 35.2 | 948 | 34.1 |  | 2.4 |
|  |  | 50,000 – 69,999 | 520 | 34.2 | 883 | 31.8 |  | 5.1 |
|  |  | 70,000 or more | 466 | 30.6 | 950 | 34.2 |  | -7.6 |
|  | Marital status^1,2^ | |  |  |  |  | 0.043 |  |
|  |  | Living without a spouse | 341 | 22.2 | 698 | 25.0 |  | -6.5 |
|  |  | Living with a spouse | 1193 | 77.8 | 2096 | 75.0 |  | 6.5 |
|  | Smoking status^1,2^ | |  |  |  |  | <0.001 |  |
|  |  | Non smoker | 540 | 35.6 | 1191 | 42.8 |  | -14.8 |
|  |  | Former smoker | 597 | 39.3 | 1043 | 37.5 |  | 3.9 |
|  |  | Current smoker | 381 | 25.1 | 551 | 19.8 |  | 12.8 |
|  | Body mass index^1,2^ | |  |  |  |  | <0.001 |  |
|  |  | Under 25 | 649 | 42.3 | 1533 | 54.8 |  | -25.0 |
|  |  | 25 – 29.9 | 655 | 42.7 | 1007 | 36.0 |  | 13.9 |
|  |  | 30 or more | 229 | 14.9 | 260 | 9.3 |  | 17.4 |
|  | Leisure-time physical activity^1,2^ | |  |  |  |  | <0.001 |  |
|  |  | Low (under 1 time per week) | 705 | 45.9 | 1109 | 39.6 |  | 12.8 |
|  |  | Moderate (1-2 time per week) | 521 | 33.9 | 1027 | 36.7 |  | -5.7 |
|  |  | Active (3 or more time per week) | 310 | 20.2 | 666 | 23.8 |  | -8.7 |
|  | Alcohol intake^1,2^ | |  |  |  |  | 0.121 |  |
|  |  | Non-drinker (under 1 intake per week) | 606 | 39.4 | 1033 | 37.0 |  | 4.9 |
|  |  | Drinker (1 or more intakes per week) | 931 | 60.6 | 1756 | 63.0 |  | -4.9 |
| **Notes:** ^1^Percentages in the column may add up to $\pm100$ because of rounding error; ^2^Missing data were excluded from the analysis – frequency missing: education level (n=36), household income (n=50), marital status (n=25), smoking status (n=50), body mass index (n=20), leisure-time physical activity (n=15), alcohol intake (n=27). | | | | | | | | |
