# Supplementary material for: Direct medical costs of cardiovascular diseases: Do cost components vary according to sex and age?
Source: PLoS One. 2024 Oct 10;19(10):e0311599. doi: 10.1371/journal.pone.0311599 (PMC11466411; doi:10.1371/journal.pone.0311599)
Supplement: Attachment — Submitted filename: Response_to_Reviewers_26August2024 HST MGO.docx [file pone.0311599.s004.docx]

**Response to reviewers**

The authors would like to thank the reviewer for very constructive comments.

Reviewer #1: Generally, this is a well-written paper using a unique dataset . A few comments below:

Title of the paper: the current title of the paper “Direct Medical Costs of Cardiovascular Diseases: Do Sex and Age Matter According to Cost Components?” does not highlight the strengths of the paper. In fact, readers may easily expect the results that age and sex matter. However, the strength of the paper lies in the difference-in-difference model with a longitudinal design and its use of a unique database. I suggest the authors reconsider the title of the paper.

Response:

Thank you for this suggestion. We agree that the title should emphasize the specific contributions of the study and we also agree that it is common knowledge that total CVD costs vary according to sex and age. However, our study makes a novel contribution to the field by showing that cost **components** differ between men and women according to age. The revised title emphasizes the novel contribution of the article as follows:

*Direct Medical Costs of Cardiovascular Diseases: Do Cost Components Vary according to Sex and Age?*

Following this comment, further emphasis has also been placed on the longitudinal design and use of a unique database in the conclusion:

*Based on a longitudinal analysis of a cohort database, our findings highlight that direct medical costs associated with a first CVD are high, especially during the first year, which emphasizes the need for primary prevention.*

Policy implications and relevance to other countries, particularly developing countries: The paper utilizes unique data that are not commonly available in lower- and middle-income countries. However, the general findings are relevant to these countries in emphasizing the need to strengthen the timeliness and quality of treatment when cardiovascular incidents occur. This can significantly reduce medical costs in subsequent years and improve the quality of life for patients.

Response:

Thank you for this suggestion, we modified the conclusion section accordingly.

Methods: The study used the case-match approach, as described in the results. The two groups are significantly different in terms of age, education, smoking behavior, BMI, and physical activity. All these factors have been documented as high-risk factors for cardiovascular diseases. The authors need to better address the possible statistical consequences and mitigating measures in the paper. (The difference-in-difference approach alone might not account for all these observed differences, as these risk factors, compounded with age, are not necessarily linear).

Response:

The aim of the difference-in-difference approach is to disentangle the costs associated with direct medical costs of CVD and related comorbidities from the costs of other (non-CVD) conditions. As suggested in the literature and explained in the methods section, every CVD case was matched with all eligible controls sharing the same sex and age to favor comparability. However, if the matching procedure was based on variables corresponding to CVD risk factors, such as BMI, the difference-in-difference analysis might have underestimated CVD costs. This is because comparing CVD cases with controls who also have the same risk factors may not accurately reflect the true costs associated with CVD, potentially leading to a skewed assessment. We added this precision in the methods section, page 8:

*The matching procedure did not include other cardiovascular risk factors, as cases and controls with similar risk profiles could have led to an underestimation of the costs associated with CVD.*

Costs: while the paper looks at the direct medical costs, but it actually examines the costs by pubic insurance, including the prescription costs. Page 6 mentioned deduction of “co-insurance amount paid by patients to compute the actual amount paid by the public health insurance system”. As a reader, I am also interested in out-of-pocket expenditures by patients on medications, as well as well OOP for other services, (if you have the data). It will be great to add those aspects to make the medical cost more complete.

Response:

As underlined in the introduction and in the methods section, the aim of this study was to estimate the direct medical costs of CVD from the economic perspective of the healthcare public system. We added this precision in the abstract.

Out-of-pocket expenditures paid by patients would be relevant to add to total costs if the study was from a societal perspective, which was not the case here.

Secondly, for patients aged 65 and older, I wonder whether there are other associated costs, such as nursing home or long-term care, or some post-operative services, and whether the authors have access to such information.

Our estimates included post-operative service costs incurred in hospitals, as this cost component is covered by the MED-ECHO database, which compiles data on stays in Quebec hospitals providing general and specialized care. Unfortunately, costs associated with nursing homes and long-term care facilities outside hospitals are not available in the existing Quebec databases. We acknowledged this limitation in the discussion section:

*Also, the costs associated with public nursing homes and long-term care establishments, other than hospitals, are not available in the MED-ECHO database. However, this likely had a limited impact on our estimates, as expenses for long-term care facilities constitute a smaller portion of total public health expenditures in Quebec (and Canada) compared to other countries (44).*

A reference (#44) has been added in the bibliography for this point: Clavet NJ, Hébert R, Michaud PC, Navaux J (2022) The Future of Long-Term Care in Quebec: What Are the Cost Savings from a Realistic Shift toward More Home Care? Canadian Public Policy, Nov 2022, vol 48 , p. 35 - 50 doi:10.3138/cpp.2022-031
