# Supplementary material for: Direct medical costs of cardiovascular diseases: Do cost components vary according to sex and age?
Source: PLoS One. 2024 Oct 10;19(10):e0311599. doi: 10.1371/journal.pone.0311599 (PMC11466411; doi:10.1371/journal.pone.0311599)
Supplement: Peer review history

# Peer review: Decision Letter 0

**DOI:** 10.1371/journal.pone.0311599.r001

**Authors:** Mabel Aoun

24 Jul 2024

PONE-D-24-10877Direct medical costs of cardiovascular diseases: do sex and age matter according to cost components?PLOS ONE

Dear Dr. Gilbert-Ouimet,

Thank you for submitting your manuscript to PLOS ONE. After careful consideration, we feel that it has merit but does not fully meet PLOS ONE’s publication criteria as it currently stands. Therefore, we invite you to submit a revised version of the manuscript that addresses the points raised during the review process.

Please provide a point-by-point response to reviewer #1 comments.

Please submit your revised manuscript by Sep 07 2024 11:59PM. If you will need more time than this to complete your revisions, please reply to this message or contact the journal office at plosone@plos.org. When you're ready to submit your revision, log on to https://www.editorialmanager.com/pone/ and select the 'Submissions Needing Revision' folder to locate your manuscript file.

Please include the following items when submitting your revised manuscript:

- A rebuttal letter that responds to each point raised by the academic editor and reviewer(s). You should upload this letter as a separate file labeled 'Response to Reviewers'.
- A marked-up copy of your manuscript that highlights changes made to the original version. You should upload this as a separate file labeled 'Revised Manuscript with Track Changes'.
- An unmarked version of your revised paper without tracked changes. You should upload this as a separate file labeled 'Manuscript'.

If you would like to make changes to your financial disclosure, please include your updated statement in your cover letter. Guidelines for resubmitting your figure files are available below the reviewer comments at the end of this letter.

If applicable, we recommend that you deposit your laboratory protocols in protocols.io to enhance the reproducibility of your results. Protocols.io assigns your protocol its own identifier (DOI) so that it can be cited independently in the future. For instructions see: https://journals.plos.org/plosone/s/submission-guidelines#loc-laboratory-protocols. Additionally, PLOS ONE offers an option for publishing peer-reviewed Lab Protocol articles, which describe protocols hosted on protocols.io. Read more information on sharing protocols at https://plos.org/protocols?utm_medium=editorial-email&utm_source=authorletters&utm_campaign=protocols.

We look forward to receiving your revised manuscript.

Kind regards,

Mabel Aoun, MD, MPH

Academic Editor

PLOS ONE

Journal Requirements:

When submitting your revision, we need you to address these additional requirements.

1. Please ensure that your manuscript meets PLOS ONE's style requirements, including those for file naming. The PLOS ONE style templates can be found at 

https://journals.plos.org/plosone/s/file?id=wjVg/PLOSOne_formatting_sample_main_body.pdf and 

https://journals.plos.org/plosone/s/file?id=ba62/PLOSOne_formatting_sample_title_authors_affiliations.pdf

2. Please provide additional details regarding participant consent. In the ethics statement in the Methods and online submission information, please ensure that you have specified what type you obtained (for instance, written or verbal, and if verbal, how it was documented and witnessed). If your study included minors, state whether you obtained consent from parents or guardians. If the need for consent was waived by the ethics committee, please include this information.

Once you have amended this/these statement(s) in the Methods section of the manuscript, please add the same text to the “Ethics Statement” field of the submission form (via “Edit Submission”).

For additional information about PLOS ONE ethical requirements for human subjects research, please refer to http://journals.plos.org/plosone/s/submission-guidelines#loc-human-subjects-research.

3. In the online submission form, you indicated that "Please send all requests for study data or materials to Mahée Gilbert-Ouimet (mahee_gilbert-ouimet@uqar.ca)."

All PLOS journals now require all data underlying the findings described in their manuscript to be freely available to other researchers, either 1. In a public repository, 2. Within the manuscript itself, or 3. Uploaded as supplementary information.

This policy applies to all data except where public deposition would breach compliance with the protocol approved by your research ethics board. If your data cannot be made publicly available for ethical or legal reasons (e.g., public availability would compromise patient privacy), please explain your reasons on resubmission and your exemption request will be escalated for approval.

4. PLOS requires an ORCID iD for the corresponding author in Editorial Manager on papers submitted after December 6th, 2016. Please ensure that you have an ORCID iD and that it is validated in Editorial Manager. To do this, go to ‘Update my Information’ (in the upper left-hand corner of the main menu), and click on the Fetch/Validate link next to the ORCID field. This will take you to the ORCID site and allow you to create a new iD or authenticate a pre-existing iD in Editorial Manager. Please see the following video for instructions on linking an ORCID iD to your Editorial Manager account: https://www.youtube.com/watch?v=_xcclfuvtxQ

5. Please include captions for your Supporting Information files at the end of your manuscript, and update any in-text citations to match accordingly. Please see our Supporting Information guidelines for more information: http://journals.plos.org/plosone/s/supporting-information.

6. Please review your reference list to ensure that it is complete and correct. If you have cited papers that have been retracted, please include the rationale for doing so in the manuscript text, or remove these references and replace them with relevant current references. Any changes to the reference list should be mentioned in the rebuttal letter that accompanies your revised manuscript. If you need to cite a retracted article, indicate the article’s retracted status in the References list and also include a citation and full reference for the retraction notice.

[Note: HTML markup is below. Please do not edit.]

Reviewers' comments:

Reviewer's Responses to Questions

**Comments to the Author**

1. Is the manuscript technically sound, and do the data support the conclusions?

The manuscript must describe a technically sound piece of scientific research with data that supports the conclusions. Experiments must have been conducted rigorously, with appropriate controls, replication, and sample sizes. The conclusions must be drawn appropriately based on the data presented. 

Reviewer #1: Yes

**********

2. Has the statistical analysis been performed appropriately and rigorously? 

Reviewer #1: Yes

**********

3. Have the authors made all data underlying the findings in their manuscript fully available?

The PLOS Data policy requires authors to make all data underlying the findings described in their manuscript fully available without restriction, with rare exception (please refer to the Data Availability Statement in the manuscript PDF file). The data should be provided as part of the manuscript or its supporting information, or deposited to a public repository. For example, in addition to summary statistics, the data points behind means, medians and variance measures should be available. If there are restrictions on publicly sharing data—e.g. participant privacy or use of data from a third party—those must be specified.

Reviewer #1: Yes

**********

4. Is the manuscript presented in an intelligible fashion and written in standard English?

PLOS ONE does not copyedit accepted manuscripts, so the language in submitted articles must be clear, correct, and unambiguous. Any typographical or grammatical errors should be corrected at revision, so please note any specific errors here.

Reviewer #1: Yes

**********

5. Review Comments to the Author

Please use the space provided to explain your answers to the questions above. You may also include additional comments for the author, including concerns about dual publication, research ethics, or publication ethics. (Please upload your review as an attachment if it exceeds 20,000 characters)

Reviewer #1: Generally, this is a well-written paper using a unique dataset . A few comments below:

Title of the paper: the current title of the paper “Direct Medical Costs of Cardiovascular Diseases: Do Sex and Age Matter According to Cost Components?” does not highlight the strengths of the paper. In fact, readers may easily expect the results that age and sex matter. However, the strength of the paper lies in the difference-in-difference model with a longitudinal design and its use of a unique database. I suggest the authors reconsider the title of the paper.

Policy implications and relevance to other countries, particularly developing countries: The paper utilizes unique data that are not commonly available in lower- and middle-income countries. However, the general findings are relevant to these countries in emphasizing the need to strengthen the timeliness and quality of treatment when cardiovascular incidents occur. This can significantly reduce medical costs in subsequent years and improve the quality of life for patients

Methods: The study used the case-match approach, as described in the results. The two groups are significantly different in terms of age, education, smoking behavior, BMI, and physical activity. All these factors have been documented as high-risk factors for cardiovascular diseases. The authors need to better address the possible statistical consequences and mitigating measures in the paper. (The difference-in-difference approach alone might not account for all these observed differences, as these risk factors, compounded with age, are not necessarily linear).

Costs: while the paper looks at the direct medical costs, but it actually examines the costs by pubic insurance, including the prescription costs. Page 6 mentioned deduction of “co-insurance amount paid by patients to compute the actual amount paid by the public health insurance system”. As a reader, I am also interested in out-of-pocket expenditures by patients on medications, as well as well OOP for other services, (if you have the data). It will be great to add those aspects to make the medical cost more complete.

Secondly, for patients aged 65 and older, I wonder whether there are other associated costs, such as nursing home or long-term care, or some post-operative services, and whether the authors have access to such information.

**********

6. PLOS authors have the option to publish the peer review history of their article (what does this mean?). If published, this will include your full peer review and any attached files.

If you choose “no”, your identity will remain anonymous but your review may still be made public.

**Do you want your identity to be public for this peer review?** For information about this choice, including consent withdrawal, please see our Privacy Policy.

Reviewer #1: **Yes: **Xiaohui Hou

**********

[NOTE: If reviewer comments were submitted as an attachment file, they will be attached to this email and accessible via the submission site. Please log into your account, locate the manuscript record, and check for the action link "View Attachments". If this link does not appear, there are no attachment files.]

While revising your submission, please upload your figure files to the Preflight Analysis and Conversion Engine (PACE) digital diagnostic tool, https://pacev2.apexcovantage.com/. PACE helps ensure that figures meet PLOS requirements. To use PACE, you must first register as a user. Registration is free. Then, login and navigate to the UPLOAD tab, where you will find detailed instructions on how to use the tool. If you encounter any issues or have any questions when using PACE, please email PLOS at figures@plos.org. Please note that Supporting Information files do not need this step.

# Author comment: Author response to Decision Letter 0

**DOI:** 10.1371/journal.pone.0311599.r002

11 Sep 2024

The authors would like to thank the reviewer for very constructive comments.

Reviewer #1: Generally, this is a well-written paper using a unique dataset . A few comments below:

Title of the paper: the current title of the paper “Direct Medical Costs of Cardiovascular Diseases: Do Sex and Age Matter According to Cost Components?” does not highlight the strengths of the paper. In fact, readers may easily expect the results that age and sex matter. However, the strength of the paper lies in the difference-in-difference model with a longitudinal design and its use of a unique database. I suggest the authors reconsider the title of the paper.

Response: 

Thank you for this suggestion. We agree that the title should emphasize the specific contributions of the study and we also agree that it is common knowledge that total CVD costs vary according to sex and age. However, our study makes a novel contribution to the field by showing that cost components differ between men and women according to age. The revised title emphasizes the novel contribution of the article as follows: 

Direct Medical Costs of Cardiovascular Diseases: Do Cost Components Vary according to Sex and Age?

Following this comment, further emphasis has also been placed on the longitudinal design and use of a unique database in the conclusion:

Based on a longitudinal analysis of a cohort database, our findings highlight that direct medical costs associated with a first CVD are high, especially during the first year, which emphasizes the need for primary prevention.

Policy implications and relevance to other countries, particularly developing countries: The paper utilizes unique data that are not commonly available in lower- and middle-income countries. However, the general findings are relevant to these countries in emphasizing the need to strengthen the timeliness and quality of treatment when cardiovascular incidents occur. This can significantly reduce medical costs in subsequent years and improve the quality of life for patients.

Response: 

Thank you for this suggestion, we modified the conclusion section accordingly.

Methods: The study used the case-match approach, as described in the results. The two groups are significantly different in terms of age, education, smoking behavior, BMI, and physical activity. All these factors have been documented as high-risk factors for cardiovascular diseases. The authors need to better address the possible statistical consequences and mitigating measures in the paper. (The difference-in-difference approach alone might not account for all these observed differences, as these risk factors, compounded with age, are not necessarily linear).

Response: 

The aim of the difference-in-difference approach is to disentangle the costs associated with direct medical costs of CVD and related comorbidities from the costs of other (non-CVD) conditions. As suggested in the literature and explained in the methods section, every CVD case was matched with all eligible controls sharing the same sex and age to favor comparability. However, if the matching procedure was based on variables corresponding to CVD risk factors, such as BMI, the difference-in-difference analysis might have underestimated CVD costs. This is because comparing CVD cases with controls who also have the same risk factors may not accurately reflect the true costs associated with CVD, potentially leading to a skewed assessment. We added this precision in the methods section, page 8:

The matching procedure did not include other cardiovascular risk factors, as cases and controls with similar risk profiles could have led to an underestimation of the costs associated with CVD. 

Costs: while the paper looks at the direct medical costs, but it actually examines the costs by pubic insurance, including the prescription costs. Page 6 mentioned deduction of “co-insurance amount paid by patients to compute the actual amount paid by the public health insurance system”. As a reader, I am also interested in out-of-pocket expenditures by patients on medications, as well as well OOP for other services, (if you have the data). It will be great to add those aspects to make the medical cost more complete.

Response: 

As underlined in the introduction and in the methods section, the aim of this study was to estimate the direct medical costs of CVD from the economic perspective of the healthcare public system. We added this precision in the abstract.

Out-of-pocket expenditures paid by patients would be relevant to add to total costs if the study was from a societal perspective, which was not the case here. 

Secondly, for patients aged 65 and older, I wonder whether there are other associated costs, such as nursing home or long-term care, or some post-operative services, and whether the authors have access to such information.

Our estimates included post-operative service costs incurred in hospitals, as this cost component is covered by the MED-ECHO database, which compiles data on stays in Quebec hospitals providing general and specialized care. Unfortunately, costs associated with nursing homes and long-term care facilities outside hospitals are not available in the existing Quebec databases. We acknowledged this limitation in the discussion section:

Also, the costs associated with public nursing homes and long-term care establishments, other than hospitals, are not available in the MED-ECHO database. However, this likely had a limited impact on our estimates, as expenses for long-term care facilities constitute a smaller portion of total public health expenditures in Quebec (and Canada) compared to other countries (44). 

A reference (#44) has been added in the bibliography for this point: Clavet NJ, Hébert R, Michaud PC, Navaux J (2022) The Future of Long-Term Care in Quebec: What Are the Cost Savings from a Realistic Shift toward More Home Care? Canadian Public Policy, Nov 2022, vol 48 , p. 35 - 50 doi:10.3138/cpp.2022-031

Attachment Submitted filename: Response_to_Reviewers_26August2024 HST MGO.docx

# Editor report: Decision Letter 1

**DOI:** 10.1371/journal.pone.0311599.r003

**Authors:** Mabel Aoun

23 Sep 2024

Direct Medical Costs of Cardiovascular Diseases: Do Cost Components Vary according to Sex and Age?

PONE-D-24-10877R1

Dear Dr. Gilbert-Ouimet,

We’re pleased to inform you that your manuscript has been judged scientifically suitable for publication and will be formally accepted for publication once it meets all outstanding technical requirements.

Within one week, you’ll receive an e-mail detailing the required amendments. When these have been addressed, you’ll receive a formal acceptance letter and your manuscript will be scheduled for publication.

An invoice will be generated when your article is formally accepted. Please note, if your institution has a publishing partnership with PLOS and your article meets the relevant criteria, all or part of your publication costs will be covered. Please make sure your user information is up-to-date by logging into Editorial Manager at Editorial Manager® and clicking the ‘Update My Information' link at the top of the page. If you have any questions relating to publication charges, please contact our Author Billing department directly at authorbilling@plos.org.

If your institution or institutions have a press office, please notify them about your upcoming paper to help maximize its impact. If they’ll be preparing press materials, please inform our press team as soon as possible -- no later than 48 hours after receiving the formal acceptance. Your manuscript will remain under strict press embargo until 2 pm Eastern Time on the date of publication. For more information, please contact onepress@plos.org.

Kind regards,

Mabel Aoun, MD, MPH

Academic Editor

PLOS ONE

Additional Editor Comments (optional):

Reviewers' comments:

# Editor report: Acceptance letter

**DOI:** 10.1371/journal.pone.0311599.r004

**Authors:** Mabel Aoun

30 Sep 2024

PONE-D-24-10877R1 

PLOS ONE

Dear Dr. Gilbert-Ouimet, 

I'm pleased to inform you that your manuscript has been deemed suitable for publication in PLOS ONE. Congratulations! Your manuscript is now being handed over to our production team.

At this stage, our production department will prepare your paper for publication. This includes ensuring the following:

* All references, tables, and figures are properly cited

* All relevant supporting information is included in the manuscript submission,

* There are no issues that prevent the paper from being properly typeset

If revisions are needed, the production department will contact you directly to resolve them. If no revisions are needed, you will receive an email when the publication date has been set. At this time, we do not offer pre-publication proofs to authors during production of the accepted work. Please keep in mind that we are working through a large volume of accepted articles, so please give us a few weeks to review your paper and let you know the next and final steps. 

Lastly, if your institution or institutions have a press office, please let them know about your upcoming paper now to help maximize its impact. If they'll be preparing press materials, please inform our press team within the next 48 hours. Your manuscript will remain under strict press embargo until 2 pm Eastern Time on the date of publication. For more information, please contact onepress@plos.org.

If we can help with anything else, please email us at customercare@plos.org.

Thank you for submitting your work to PLOS ONE and supporting open access. 

Kind regards, 

PLOS ONE Editorial Office Staff

on behalf of

Dr. Mabel Aoun 

Academic Editor

PLOS ONE
